# Telephone calls to boost response rates in the collection of outcome data

**DOI:** 10.1186/1745-6215-14-S1-P79

**Published:** 2013-11-29

**Authors:** Suzanne Breeman, Seonaidh Cotton, Alison McDonald, Gladys McPherson, Graeme MacLennan, Hanne Bruhn

**Affiliations:** 1 University of Aberdeen, Aberdeen, UK

## 

Using telephone calls in the context of randomised controlled trials (RCTs) is not a new idea. There are a number of ways in which telephone calls can be used, including, for example: as a precursor to sending questionnaires or reminder questionnaires (to let the participant know to expect a questionnaire), as a follow-up reminder to participants who have not completed postal questionnaires, to collect primary outcome data when a postal questionnaire has not been returned, or to replace postal questionnaires. There is, however, limited data available in terms of the implications and impact of such telephone calls.

We will present data for different applications of telephone calls, across a number of different RCTs in different disease areas and in different study populations. Our data suggests that using telephone calls either as precursors to a questionnaire, or as a reminder, can increase the return rate. There can also be added value from a telephone call if it is possible to collect primary outcome data as part of the call, and we will present examples of this. There is, however, resource implications in terms of the number of telephone calls required to achieve contact with the participant, as well as ethical issues around securing approval for such calls.

